# The NDR/LATS Kinase Cbk1 Controls the Activity of the Transcriptional Regulator Bcr1 during Biofilm Formation in *Candida albicans*

**DOI:** 10.1371/journal.ppat.1002683

**Published:** 2012-05-10

**Authors:** Pilar Gutiérrez-Escribano, Ute Zeidler, M. Belén Suárez, Sophie Bachellier-Bassi, Andrés Clemente-Blanco, Julie Bonhomme, Carlos R. Vázquez de Aldana, Christophe d'Enfert, Jaime Correa-Bordes

**Affiliations:** 1 Departamento Ciencias Biomédicas, Universidad de Extremadura, Badajoz, Spain; 2 Institut Pasteur, Unité Biologie et Pathogénicité Fongiques, Département Génomes et Génétique, Paris, France; 3 INRA, USC2019, Paris, France; 4 Instituto de Biología Funcional y Genómica, CSIC-Universidad de Salamanca, Salamanca, Spain; 5 Cell Cycle Group, MRC Clinical Sciences Centre, Imperial College, London, United Kingdom; UCSF, United States of America

## Abstract

In nature, many microorganisms form specialized complex, multicellular, surface-attached communities called biofilms. These communities play critical roles in microbial pathogenesis. The fungal pathogen *Candida albicans* is associated with catheter-based infections due to its ability to establish biofilms. The transcription factor Bcr1 is a master regulator of *C. albicans* biofilm development, although the full extent of its regulation remains unknown. Here, we report that Bcr1 is a phosphoprotein that physically interacts with the NDR kinase Cbk1 and undergoes Cbk1-dependent phosphorylation. Mutating the two putative Cbk1 phosphoacceptor residues in Bcr1 to alanine markedly impaired Bcr1 function during biofilm formation and virulence in a mouse model of disseminated candidiasis. Cells lacking Cbk1, or any of its upstream activators, also had reduced biofilm development. Notably, mutating the two putative Cbk1 phosphoacceptor residues in Bcr1 to glutamate in *cbk1*Δ cells upregulated the transcription of Bcr1-dependent genes and partially rescued the biofilm defects of a *cbk1*Δ strain. Therefore, our data uncovered a novel role of the NDR/LATS kinase Cbk1 in the regulation of biofilm development through the control of Bcr1.

## Author Summary

*C. albicans* infections frequently involve the formation of biofilms on implanted devices such as indwelling catheters. These complex communities of surface-associated fungal cells embedded in a matrix of extracellular polysaccharides protect *C. albicans* from host defences and antifungal agents. In recent years, several genes involved in the development of biofilms of *C. albicans* have been identified. These studies have uncovered complex regulatory networks that control the activity of several transcription factors during different steps of biofilm development. Bcr1 is a transcription factor that plays a major role in this process and yet, its regulation has not been studied extensively. Here, we show that Bcr1 function in biofilm formation and virulence requires phosphorylation of threonine 191 and serine 556 by the NDR/LATS kinase Cbk1. Moreover, given that Cbk1 is also required for the onset and maintenance of hyphal growth, our study highlights this kinase as a pivotal regulator of several developmental programs that are essential for the biology and pathogenesis of *C. albicans*.

## Introduction

Biofilms are surface-attached microbial communities embedded in an extracellular matrix. Cells in a biofilm exhibit phenotypic properties different from those of their planktonic counterparts, including an increased resistance to the host immune system and to antimicrobial agents [1]–[3]. Several tissues, as genitourinary or oral epithelia, and biomedical devices can serve as substrates for biofilm development. In this context, biofilm formation is a key feature in microbial pathogenesis.

Among the pathogenic fungi, *C. albicans* is one of the organisms most commonly associated with implant-related infections [2], [4], [5]. *C. albicans* is a polymorphic fungus that can change between three different forms: yeast, pseudohyphae and hyphae. Morphogenetic transitions are critical for the acquisition of proper biofilm architecture: initially, a basal layer of cells is formed when yeast cells attach to a surface followed by cell division and proliferation. In a second phase, cells differentiate into hyphal and pseudohyphal forms and produce extracellular material; the development of these forms and the increase in extracellular matrix deposition would finally arise in a dense and mature biofilm structure. Genes required for hyphal morphogenesis, cell wall remodeling, amino acid and lipid metabolism and glycolytic processes have been involved in the progression of biofilm formation in *C. albicans* (for a review, see [6]). Notably, biofilm development requires the activation of specific transcription programs different from those of free-living planktonic cells [7]. Tec1, a hypha-specific gene regulator; Bcr1, required for the expression of different cell wall proteins and Zap1, which governs matrix production, are examples of *C. albicans* biofilm transcriptional regulators [8]–[11]. In particular, Bcr1 has been shown to regulate the expression of a subset of genes encoding cell wall-anchored proteins including members of the agglutinin-like protein family, such as Als1 and Als3, and the hyphal wall protein Hwp1 [9], [12]. Deletion of *BCR1* results in defective biofilm formation *in vivo* and *in vitro* because of altered cell-to-cell interactions mediated by Als1, Als3 and Hwp1 [9].

The RAM signaling network is a conserved pathway that controls cell separation, polarized growth and cell integrity in yeast [13]–[17]. In *Saccharomyces cerevisiae*, the central core of the pathway consists of the Cbk1 kinase, a member of the NDR/LATS kinase family, its binding partner Mob2, the scaffolding protein Tao3, and the Ste20-like kinase Kic1. The activity of Cbk1, the main effector of the RAM pathway, is regulated by phosphorylation in a Kic1- and Tao3-dependent manner [18]. While Cbk1 polarity targets still remain largely unknown, the control of cell separation depends on the regulation of the transcription factor Ace2 [14], [19], [20]. It has been shown that Cbk1 phosphorylates Ace2 at the end of mitosis, triggering its accumulation at the daughter cell nucleus and leading to the asymmetric expression of genes involved in septum degradation [20]. Orthologues of the RAM pathway have been recently reported in *C. albicans* [16], [17]. As in *S. cerevisiae*, RAM mutants exhibited cell separation defects and loss of cell polarity. In addition, the phenotypic analysis of a library of protein kinase mutant strains has shown that a *cbk1Δ* mutant is defective in biofilm formation although the function of Cbk1 in this process has not been further characterized [21].

The protein Cbk1 is a basophilic S/T protein kinase that exhibits a high specificity for motifs with His at the −5 position and the basic amino acids Lys or Arg at either the −3 or −2 positions [20]. This motif also seems to be the substrate of other kinases of the NDR/LATS family, such as the *Drosophila melanogaster* Warts/Lats and human LATS1 [22]–[24]. A search for this motif in the *C. albicans* proteome revealed that 0.6% of the proteins contain two or more putative Cbk1 consensus sites. Consistent with the conserved role of NDR kinases in the control of transcriptional activity, transcription factors were enriched among the putative targets, including several involved in biofilm development such Ace2 (3 sites), Bcr1 (2 sites), Nrg1 (3 sites) and Zap1 (3 sites) [25], [8], [10], [26]. Therefore, this observation suggested that the NDR/LATS kinase Cbk1 could regulate biofilm formation by acting on transcription factors that trigger the biofilm developmental program in *C. albicans*. In the present study, we focus on the role of Cbk1 in the control of Bcr1, a master regulator of biofilm formation [27]. We and others previously established that Cbk1 is essential for hyphal development [16], [17]. Here, we demonstrate that Cbk1 is also a regulator of biofilm formation through phosphorylation of Bcr1 at T191 and S566 residues. Thus, the Cbk1 kinase is emerging as a pivotal regulator of several developmental programs that are essential for the biology and pathogenesis of *C. albicans*.

## Results

### RAM mutants show severe defects in biofilm formation *in vitro*

**Figure 1:**
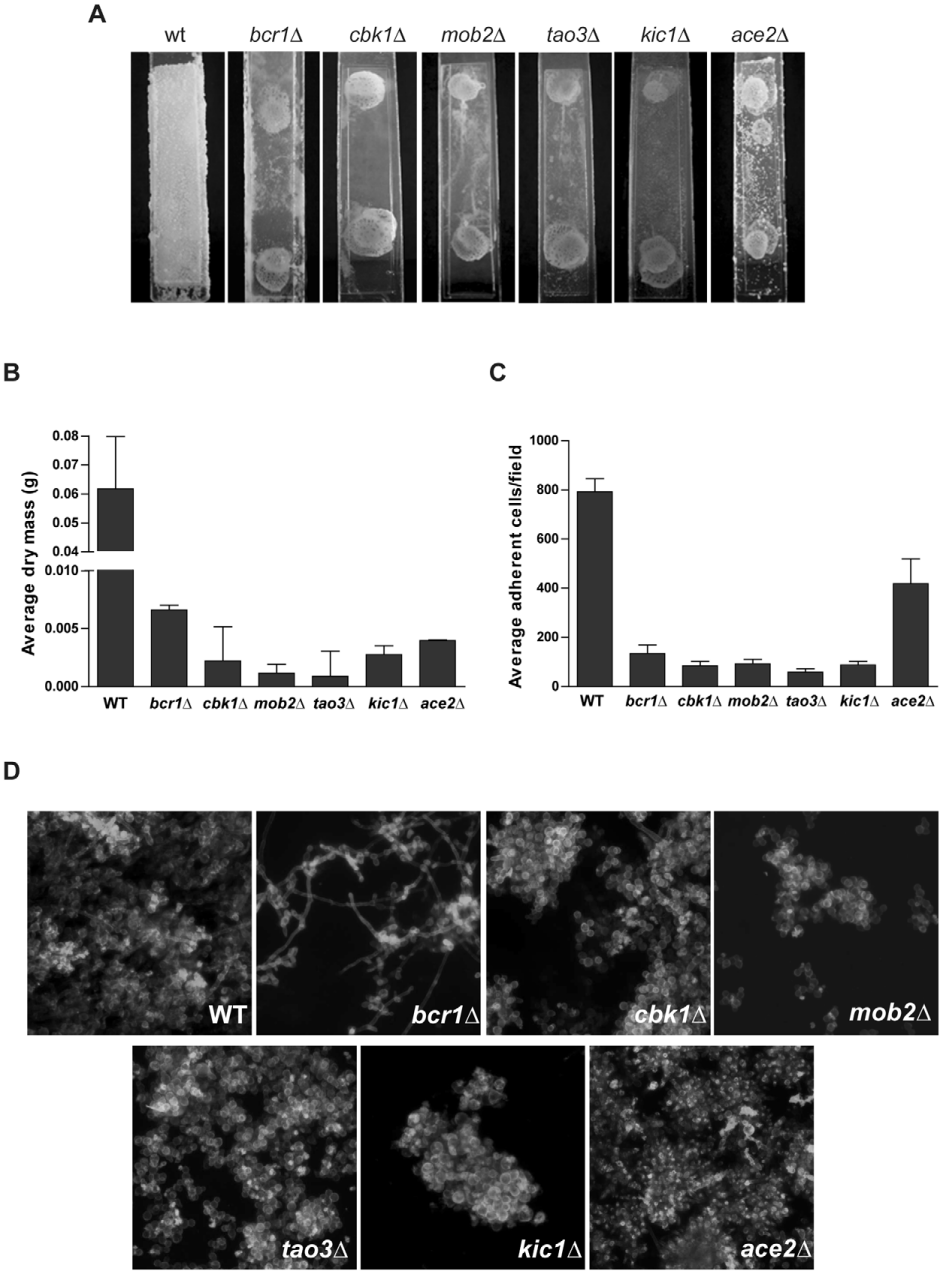
The RAM pathway is required for biofilm formation *in vitro*. (A) The wild-type (CEC369) and *bcr1*Δ (JC1081) reference strains, *cbk1*Δ (JC1080), *mob2*Δ (JC525), *tao3*Δ (JC848), *kic1*Δ (JC798) and *ace2*Δ (JC343) mutants were incubated during 40 hours in biofilm-inducing conditions on Thermanox plastic slides in microfermentors. (B) Determination of biofilm dry mass collected from microfermentors shown in (A). Average results of two independent experiments done in duplicate are shown. Error bars represent the standard deviation of the data throughout the paper, unless otherwise indicated. (C) Adherence to plastic slides of WT, *bcr1Δ*, RAM mutants and *ace2*Δ, as detailed in Materials and Methods. The results shown are the mean of 3 independent experiments counting 30 fields per strain in each one. (D) Biofilms induced on silicon squares for 60 hours were stained with calcofluor white and concanavaline A for CSLM visualization.

Although a role for Cbk1 in biofilm formation has been reported [21], no quantification of biofilm-formation defect of cells lacking Cbk1 has been published. To characterize the severity of the phenotype of *cbk1Δ* cells in biofilm formation, cells were grown under biofilm inducing conditions using a microfermentor model [7] and compared to wild-type and *bcr1Δ* reference strains. In the wild-type strain, cells thoroughly colonized the Thermanox plastic slide producing a thick and cottony biofilm. In contrast, *cbk1Δ* mutant developed very poor haze-like biofilm structures in which colonization of the plastic slides and biomass production were dramatically reduced, similar to that of *bcr1Δ* cells (Figure 1A and 1B). The function of Cbk1 in biofilm formation depends on the RAM pathway since mutants defective in different components of the RAM network (*kic1*Δ, *tao3*Δ, and *mob2*Δ) phenocopied *cbk1Δ* cells. The same strains were tested using a biofilm formation model on silicon squares [8] to perform confocal scanning laser microscopy (CSLM). As expected, wild-type reference strain produced a complex biofilm structure of interconnected yeasts, pseudohyphae and hyphae whereas *bcr1Δ* mutant developed a rudimentary biofilm that included yeast cells with few pseudophyphae and hyphae. However, RAM mutants produced biofilms comprised exclusively of isolated clumps of yeast cells (Figure 1D). Thus, our data indicates that the absence of RAM signaling causes a severe defect in biofilm development.

The structural defects of RAM mutant biofilms could be due to their inability to develop hyphae [16], [17]. Nevertheless, in contrast to other mutants defective in hyphal formation such as *cph1*Δ *efg1*Δ [7], RAM mutants were even unable to form a compact primary layer of yeast cells on the slides, which might suggest a defect in adhesion. The RAM pathway regulates the activity of the transcription factor Ace2, which controls the expression of cell wall genes [19], [28], [15]. As shown in Figure 1, no significant differences were found in biomass production in microfermentor experiments between *ace2Δ* and RAM mutants. It has been reported that the *ace2*Δ mutant has a moderate defect in adherence to plastic surfaces [25]. In order to assess whether the defects in attachment to plastic or silicone surfaces of the RAM mutants were due to an Ace2 misregulation, the adherence of RAM and *ace2*Δ mutants was quantified and compared to wild-type and *bcr1Δ* reference strains (Figure 1C; see Materials and Methods). Whereas *ace2*Δ cells showed a 50% reduction in adherence compared to the wild-type control, the defect exhibited by RAM mutants was more dramatic, with a 90% decrease in the number of adherent cells similar to that of *bcr1Δ* cells. Therefore, these results indicate that adherence impairments of RAM mutants could not be solely due to defects in Ace2 transcriptional regulation.

### Bcr1 is a phosphoprotein that interacts with Cbk1 *in vivo*

**Figure 2:**
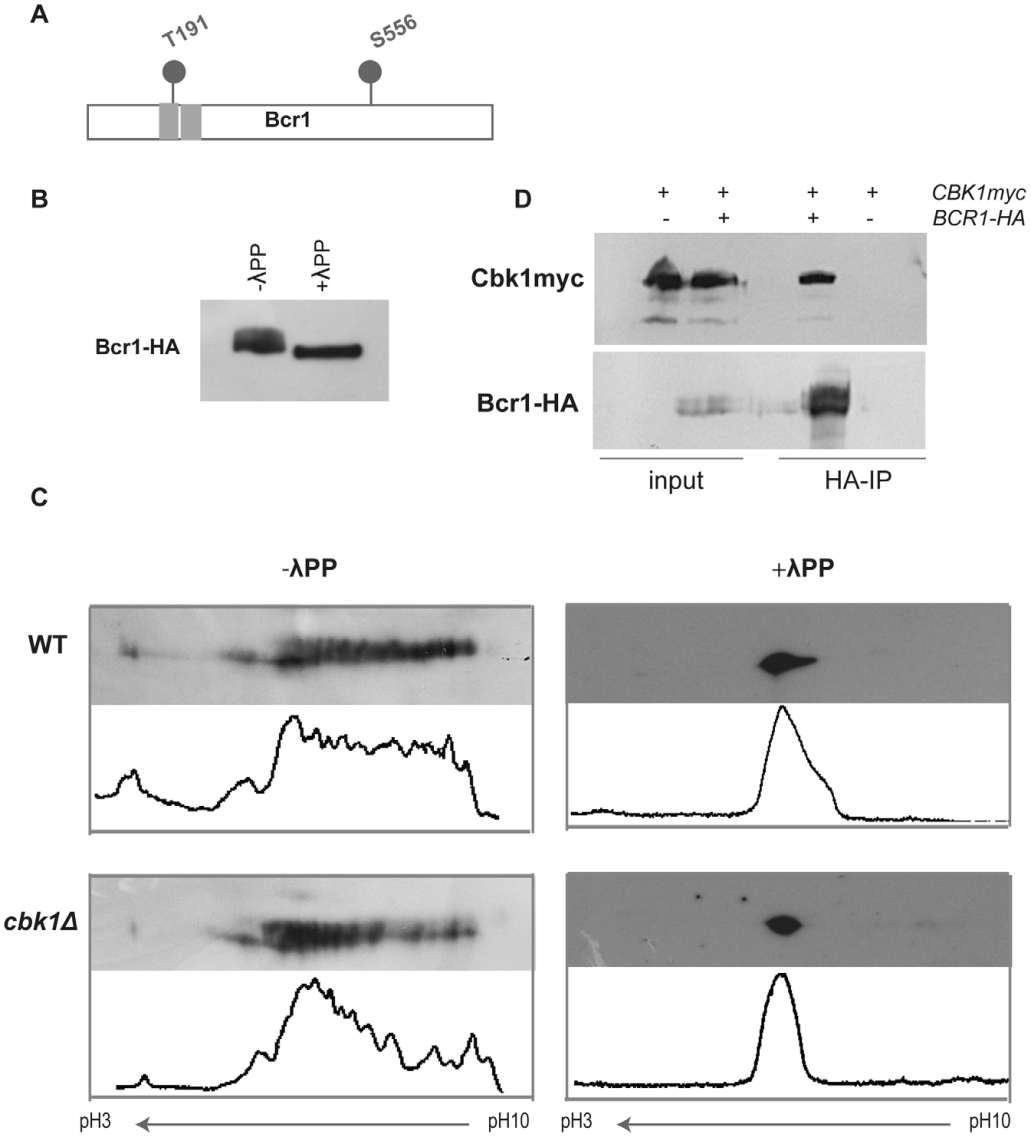
Bcr1 is a phosphoprotein that interacts *in vivo* with Cbk1. (A) Schematic representation of Cbk1 consensus phosphorylation sites in Bcr1. Gray rectangles: Zn finger domains. (B) Cell extracts from a *BCR1-HA* strain (JC1144) grown at 37°C in Spider medium were analyzed by Western blot using anti-HA antibodies. A fraction from the same lysates was treated with λ-phosphatase (λPP). (C) Cell lysates from wild-type (WT, JC1144) and *cbk1*Δ (JC1159) strains grown in Spider medium at 37°C were subjected to 2D-WB and probed with anti-HA antibodies. Half of each lysate was treated with λ-phosphatase. Histograms obtained using ImageJ show the quantification of the relative intensity of each spot of the blot. (D) Protein extracts from a *CBK1*-*myc BCR1*-*HA* strain (JC1151) were immunoprecipitated using anti-HA antibodies. A strain carrying *CBK1-myc* (JC305) was used as negative control of the immunoprecipitation. Samples were separated by SDS-PAGE and probed with anti-myc or anti-HA antibodies.

The above results suggested that the Cbk1/Mob2 complex, the main effector of the RAM pathway, might target key regulators of biofilm formation other than Ace2. The transcription factor Bcr1 controls the expression of cell-surface genes and it is required for biofilm formation *in vitro* and *in vivo* [8], [9]. Interestingly, Bcr1 contains two putative Cbk1 consensus phosphorylation sites at T191 and S556, one of which is located at the end of the first Zn finger (Figure 2A), suggesting that it could be regulated by the RAM pathway. Western blot analysis of cells expressing a functional Bcr1 tagged allele (*BCR1-HA*) grown at 37°C in Spider medium showed that Bcr1 is a phosphoprotein (Figures 2B). To analyze the phosphorylation pattern in greater detail, we used two-dimensional gel electrophoresis followed by Western blotting (2D-WB). On the basis of this approach, Bcr1 showed a complex phosphorylation pattern with multiple spots along the pH gradient (Figure 2C). Treatment with λ-phosphatase produced a single dot that ran at the middle of the pH gradient. When cell extracts from the *cbk1*Δ strain were analyzed by 2D-WB, the relative abundance of the different Bcr1 isoforms was shifted to intermediate spots, corresponding to less phosphorylated isoforms (Figure 2C). Thus, the phosphorylation state of Bcr1 appears to be Cbk1-dependent.

To obtain further evidence that Bcr1 interacts with Cbk1 *in vivo*, we performed co-immunoprecipitation experiments from extracts of cells expressing Cbk1-myc and Bcr1-HA from their native promoters. This approach clearly showed an *in vivo* interaction between these two proteins (Figure 2D). Taken together, these data suggest that Bcr1 could be a direct target of the NDR/LATS kinase Cbk1 *in vivo*.

### The Cbk1 consensus phosphorylation sites in Bcr1 are required for adherence and biofilm formation *in vitro*

**Figure 3:**
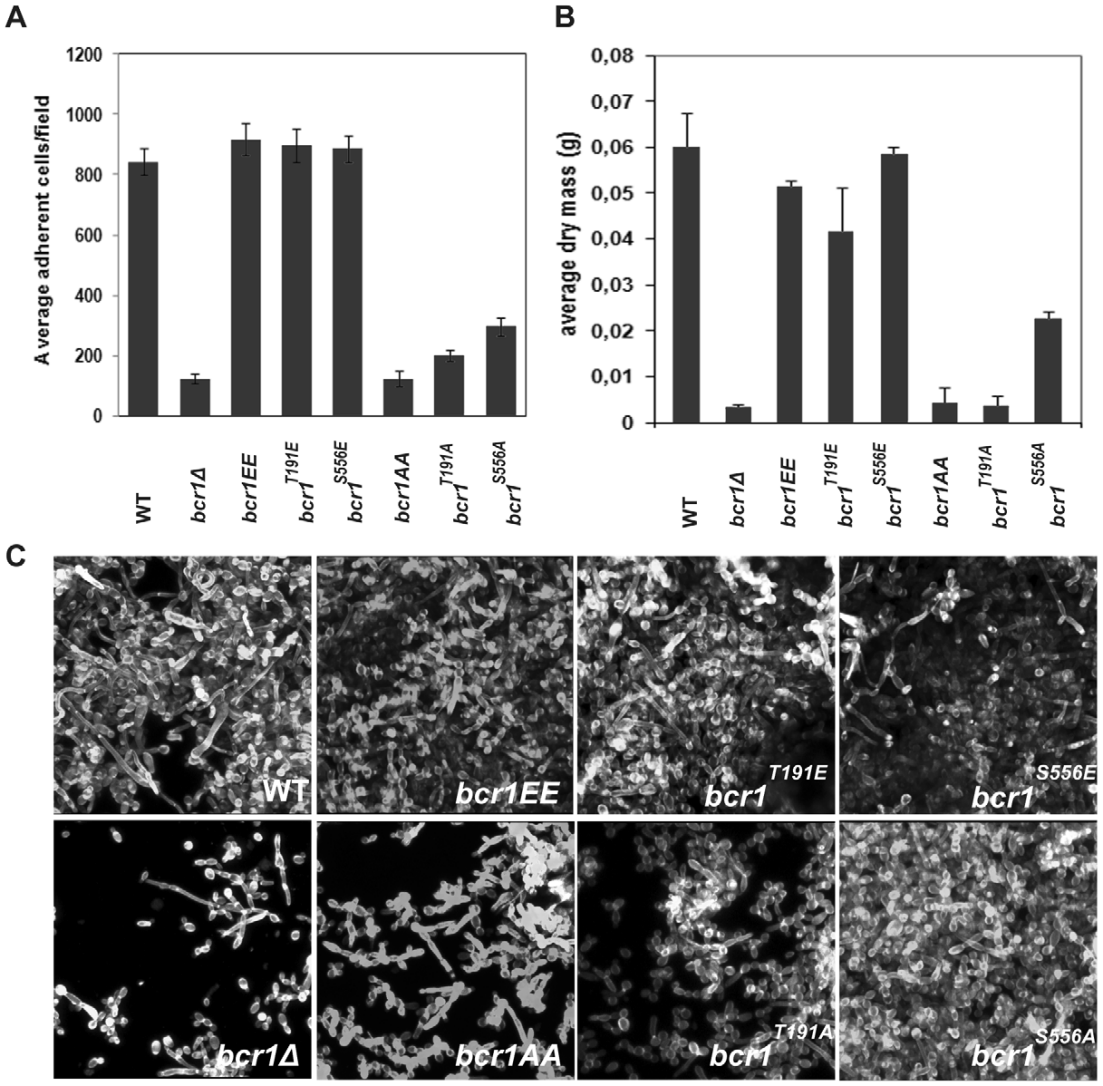
Characterization of the phenotype of *BCR1*-phosphorylation mutants in biofilm formation *in vitro*. *BCR1* phosphomimetic (*bcr1* ^T191E^/*bcr1*Δ, JC1094; *bcr1* ^S556E^/*bcr1*Δ, JC1092; *bcr1EE*/*bcr1*Δ, JC1180) and phosphodefective (*bcr1* ^T191A^/*bcr1*Δ, JC1093; *bcr1* ^S556A^/*bcr1*Δ, JC1088; *bcr1AA*/*bcr1*Δ, JC1178) mutants were grown in biofilm-inducing conditions for 40 hours using microfermentors. *BCR1/bcr1*Δ (WT, JC1089) and *bcr1*Δ (JC1081) strains were used as controls. (A) Quantification of adherent cells. (B) Biofilm dry mass determination. (C) Biofilms grown using silicon squares models were stained with calcofluor white for CSLM visualization.

To determine whether the putative Cbk1 phosphorylation sites are important for Bcr1 function *in vivo*, we mutated them and analyzed the effect of these mutations in biofilm formation. We constructed phosphomimetic and phosphodefective versions of Bcr1 by replacing the T191 and S556 putative Cbk1 phosphoacceptor residues with Glu or Ala, respectively. The mutant alleles were used to replace the wild-type copy of a heterozygous *BCR1/bcr1*Δ strain, resulting in strains containing the mutant alleles under the control of the native promoter as the sole source of Bcr1 in the cell. The phenotypes of these *bcr1* mutants were compared to those of the *BCR1* heterozygous and *bcr1*Δ null mutant strains. It has been reported that *bcr1*Δ mutants exhibit severe defects in adherence and biofilm formation [8], [9]. We did not observe any significant difference in adherence between the *bcr1^T191E^*, *bcr1^S556E^* and *bcr1^T191E/S556E^* (referred to as *bcr1EE*) mutant strains and the *BCR1/bcr1*Δ reference strain (Figure 3A). Furthermore, biofilm biomass production by the three phosphomimetic mutants was similar to that of the reference wild-type control and no significant statistical differences were found between them (*bcr1^T191E^*, p = 0.0826; *bcr1^S556E^*, p = 0.7472 and *bcr1^T191E/S556E^*, p = 0.1282) (Figure 3B). In addition, CSLM imaging revealed that biofilm architecture of these mutants was similar to that of the wild type (Figure 3C). In contrast, all strains carrying mutations to alanine (*bcr1^T191A^, bcr1^S556A^* and *bcr1^T191A/S556A^* referred to as *bcr1AA*) showed a significant decrease in the number of adherent cells and in biofilm development. These defects were more severe in the *bcr1^T191A^* and *bcr1AA* mutants, which showed values closer to the *bcr1*Δ mutant, than in the *bcr1^S556A^* mutant. Finally, CSLM imaging revealed that the biofilm structure of alanine mutants was very rudimentary and mainly composed of yeast cells, with few pseudohyphae and hyphae. However, the *bcr1^S556A^* mutant exhibited a more organized and compact primary cell layer than that formed by the *bcr1^T191A^* and *bcr1AA* strains, suggesting a different contribution of the two phosphorylation sites to Bcr1 regulation. Thus, these data indicate that the Cbk1 phosphorylation sites in Bcr1 are physiologically relevant for Bcr1 function during biofilm formation.

### Cbk1-mediated phosphorylation of Bcr1 is required for full *ALS3 and ALS1* expression

**Figure 4:**
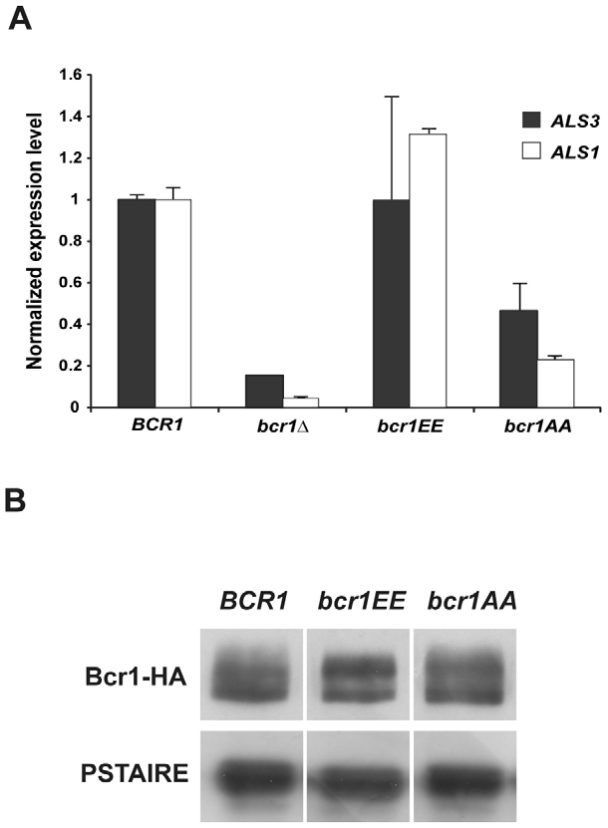
Phosphorylation of Cbk1 consensus sites is required for Bcr1 transcriptional activity. (A) Expression of the Bcr1 target genes *ALS3* and *ALS1* measured by quantitative RT-PCR in the wild-type (JC1089), *bcr1*Δ (JC1081), *bcr1EE* (JC1180) and *bcr1AA* (JC1178) strains normalized using *ADE2*. The data are the mean of two independent experiments. (B) Cells from the wild-type *BCR1-HA* (JC1144), *bcr1EE-HA* (JC1177) and *bcr1AA-HA* (JC1176) strains were grown at 37°C in Spider medium. Protein extracts were probed with anti-HA antibodies. Anti-PSTAIRE antibodies were used as loading control.

To determine whether Cbk1 phosphorylation sites in Bcr1 were important to regulate its transcriptional activity, we analyzed the expression levels of Bcr1-dependent genes in cells containing the phosphomutant Bcr1 proteins. Figure 4A shows *ALS3* and *ALS1* transcript levels measured by RT-PCR in the wild-type, *bcr1*Δ, *bcr1EE* and *bcr1AA* strains. Both *ALS3* and *ALS1* expression in *bcr1EE* cells was similar to that of the wild-type strain, whereas a significant reduction was observed in the *bcr1AA* mutant. Nevertheless, expression was still significantly higher in the *bcr1AA* mutant compared to the *bcr1*Δ null mutant, in which the transcripts were dramatically reduced, as previously described [8], [9]. The decrease in the expression of these Bcr1-dependent genes observed in *bcr1AA* cells was not due to changes in Bcr1 abundance, since Bcr1 protein levels were similar in the *bcr1AA-HA*, *bcr1EE-HA* and *BCR1-HA* strains (Figure 4B). Therefore, these findings suggest that phosphorylation of Bcr1 at Cbk1 phospho-acceptor sites is important for full expression of Bcr1-dependent genes *ALS3* and *ALS1*.

### The *bcr1EE* allele partially complements the *cbk1*Δ biofilm defect *in vitro*

**Figure 5:**
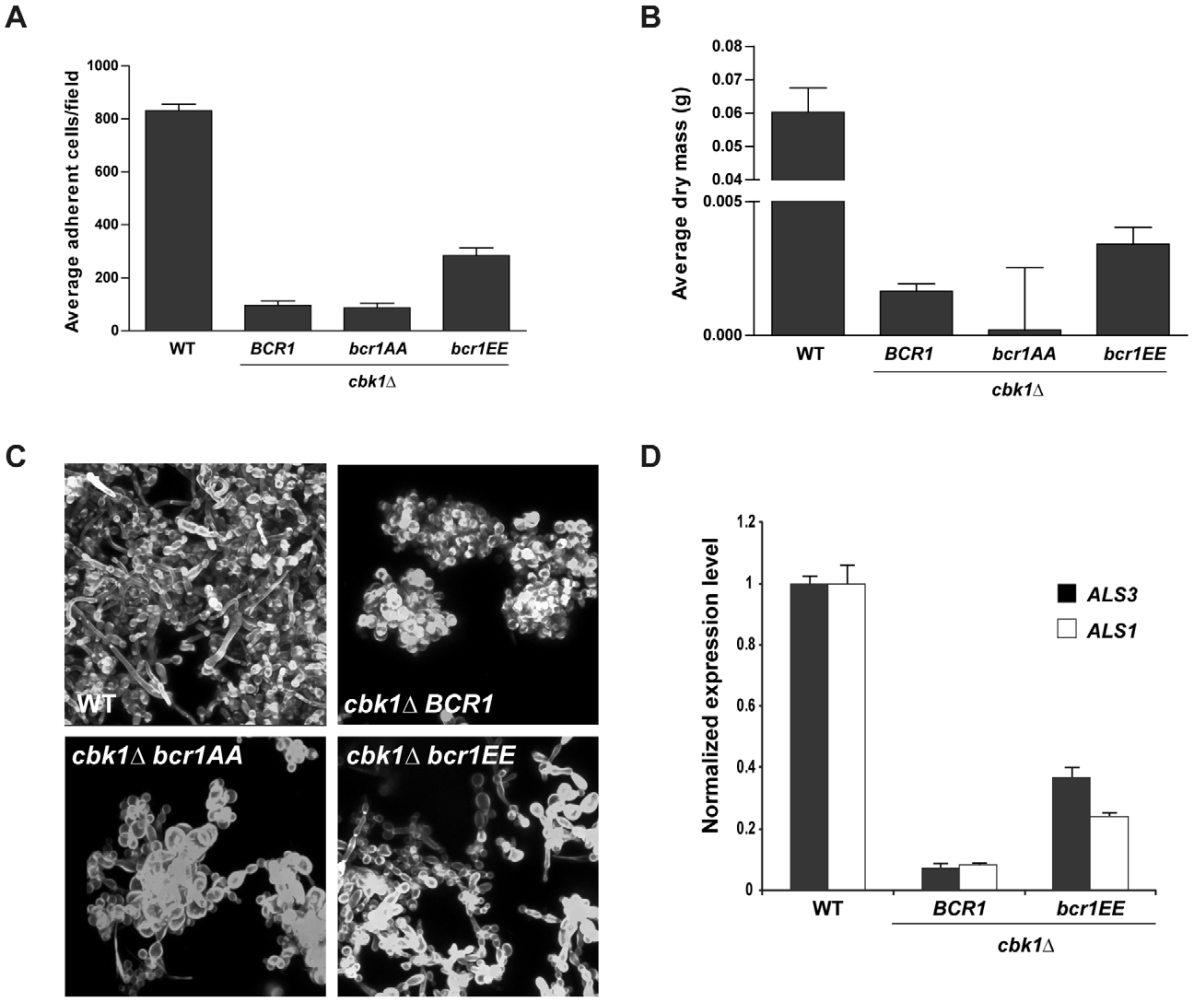
Characterization of the *in vitro* biofilm formation phenotype of the *cbk1*Δ *bcr1EE* double mutant. (A) Quantification of adherence ability to plastic slides of wild-type (JC1089), *cbk1*Δ *BCR1* (JC1082), *cbk1*Δ *bcr1AA* (JC1096) and *cbk1*Δ *bcr1EE* (JC1099) cells. (B) The same strains were incubated during 40 hours in microfermentors and biofilm dry mass was determined. (C) CSLM images of biofilms induced using silicone squares after calcofluor white staining. (D) Quantitative RT-PCR measurements of *ALS3* and *ALS1* transcription levels in the wild-type, *cbk1*Δ *BCR1* and *cbk1*Δ *bcr1EE* strains normalized using *ADE2*.

The hypothesis stated above predicts that the *bcr1EE* allele might suppress, at least partially, the biofilm defects observed in a *cbk1*Δ strain. We tested this prediction by integrating the *bcr1EE* or *bcr1AA* alleles in a *cbk1*Δ*/cbk1*Δ *BCR1*/*bcr1*Δ background. The adherence ability and biomass production of the double mutants were tested and compared to those of the parental strain. While the results obtained with the *cbk1*Δ *bcr1AA* mutant were similar to those found for the *cbk1*Δ reference strain, the introduction of *bcr1EE* in the *cbk1*Δ background resulted in a significant increase in the number of adherent cells and biomass (Figure 5A and 5B). The *cbk1*Δ *bcr1EE* double mutant exhibited adherence values 3-fold-higher than those of the *cbk1*Δ mutant and biofilm biomass production was doubled, although these values were still lower than those found in the wild type. CSLM imaging also revealed qualitative differences between *cbk1*Δ *bcr1EE* and *cbk1*Δ strains (Figure 5C). Whereas the rudimentary biofilms formed by the *cbk1*Δ strain were composed of few groups of round yeast cells, the *cbk1*Δ *bcr1EE* strain produced a biofilm with higher cell density that included yeast and pseudohyphal cells. Therefore, the phosphomimetic *bcr1EE* mutant partially rescues the biofilm formation defects of the *cbk1*Δ strain.

Given the phenotypic differences between both strains, we next analyzed whether the introduction of *bcr1EE* allele had any effect on Bcr1 transcriptional activity. RT-PCR assays showed that *ALS3* and *ALS1* expression levels in the *cbk1*Δ mutant were dramatically decreased compared to wild-type levels (Figure 5D). Notably, the *cbk1*Δ *bcr1EE* double mutant exhibited a significant increase in *ALS3* and *ALS1* transcripts versus the *cbk1*Δ reference strain (5.3-fold increase for *ALS3* and 3-fold increase for *ALS1*). Altogether, these results strongly suggest that the RAM pathway regulates Bcr1 transcriptional activity and, therefore, biofilm development, through Cbk1-dependent phosphorylation.

### Analysis of Bcr1 phosphomutants in disseminated murine candidiasis

**Figure 6:**
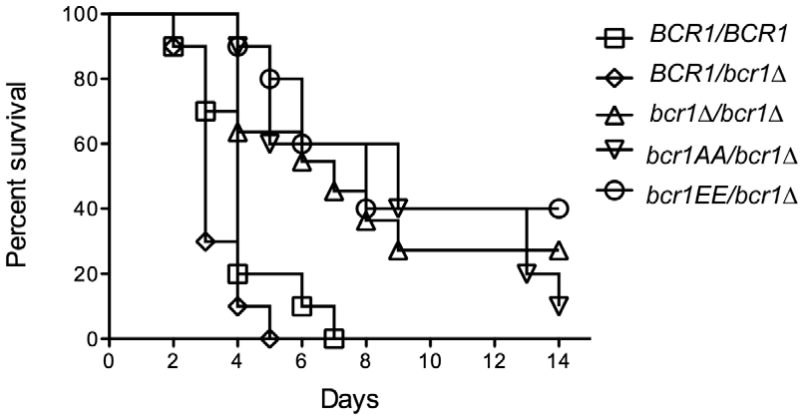
Cbk1-dependent phosphorylation of Bcr1 is required for virulence during disseminated candidiasis. Mice were infected intravenously with 5×10^5^ yeast phase cells of the indicated *C. albicans* strains. The graphs represent the survival over a 14-day period (n = 10 mice per strain). * P<0.005.

In addition to its role in biofilm formation, Bcr1 has been shown to contribute to *C. albicans* virulence. Indeed, inactivation of *BCR1* results in a fitness defect in a mouse model of disseminated candidiasis when compared to the wild-type and other *C. albicans* knock-out mutant strains [29]. Hence, we tested whether the Cbk1-dependent phosphorylation of Bcr1 could contribute to its function in virulence. Immuno-competent BALB-c mice were infected intravenously with *C. albicans BCR1/BCR1*, *BCR1/bcr1Δ*, *bcr1Δ/bcr1Δ, bcr1AA* and *bcr1EE* strains and mortality was recorded over a 14-day period. As shown in Figure 6, inactivation of one copy of *BCR1* did not impair *C. albicans* virulence while inactivation of the two *BCR1* alleles resulted in significant reduction of *C. albicans* virulence (p<0.005), as anticipated from previous observations [29]. Similarly, the *bcr1AA* mutant showed reduced virulence, suggesting that Cbk1-mediated phosphorylation of Bcr1 is required for *C. albicans* virulence. The most striking result was obtained with the *bcr1EE* mutant that showed reduced virulence to a level similar to that observed with the *bcr1Δ* and *bcr1AA* mutants (Figure 6). These results suggest that phosphorylation and dephosphorylation of Bcr1 is required for full virulence of *C. albicans*.

## Discussion

Control of gene expression is a conserved function of NDR/LATS kinases in eukaryotic cells [30], [31]. The aim of this work was to assess whether the NDR/LATS kinase Cbk1 was involved in transcriptional control during *C. albicans* biofilm development. A survey for Cbk1 consensus sites in the *C. albicans* proteome allowed the identification of a subset of C_2_H_2_ Zn finger transcriptional regulators of biofilm development as putative Cbk1 targets (Bcr1, 2 sites; Ace2, 3 sites; Nrg1, 3 sites; and Zap1, 3 sites). Since these proteins are required at different stages of biofilm formation [25], [8], [10], [26], this observation might suggest that Cbk1 regulates transcriptional activity at different steps during *C. albicans* biofilm development. Here, we have studied the function of Cbk1 at early stages of biofilm formation, and our findings suggest that full activation of Bcr1 is dependent on Cbk1 phosphorylation.

Recently, a phenotypic analysis of a collection of protein kinase mutants identified Cbk1 as a kinase required for biofilm formation [21]. In agreement with this observation, we have found that RAM mutants have severe biofilm formation defects similar to that of the *cbk1Δ* strain. The impaired biofilm development of RAM mutants could be due to their inability to develop hyphae [32], [16], [17], since hyphal formation is important for biofilm development in *C. albicans* [33]. However, a *cph1Δ efg1Δ* strain defective in hyphae development is able to produce rudimentary biofilms and express a set of biofilm-related genes in a microfermentor model [7]. In contrast to *cph1Δ efg1Δ* cells, RAM mutants did not produce a compact layer of yeast cells in the same biofilm-formation model, suggesting a severe defect in cell-surface adherence. Given the central role of Bcr1 in this process, we rationalized that Cbk1 phosphorylation could be required for Bcr1 activation.

The results presented in this report indicate that Cbk1 regulates Bcr1 function during biofilm development, and this is based on the following evidences. First, biofilm mass production and adherence of RAM mutants were similar to that of *bcr1Δ* cells. Second, using 2D gels we have shown that the Bcr1 phosphorylation status was partially Cbk1-dependent. In addition, coimmunoprecipitation experiments revealed a physical interaction between Cbk1 and Bcr1. Third, the *bcr1AA* allele lacking Cbk1 phosphorylation sites phenocopied *bcr1Δ* cells, whereas the *bcr1EE* allele behaved as the wild-type, indicating that phosphorylation of the two Cbk1-consensus sites is essential for Bcr1 function. In addition, the expression of Bcr1-dependent genes were reduced in *bcr1AA* cells as compared to the *BCR1/bcr1Δ* strain, suggesting that phosphorylation of Cbk1 sites in Bcr1 is required for full activation of the transcriptional program required for biofilm formation. In support of this idea, an upregulation in *ALS3* and *ALS1* expression was observed in the *bcr1EE cbk1Δ* strain in comparison to the *cbk1Δ* mutant (5.3-fold increase for *ALS3* and 3-fold increase for *ALS1*). Given that overexpression of these adhesins in a *bcr1Δ* background restores biofilm formation *in vitro* and *in vivo* [9], [34], the downregulation of *ALS3* and *ALS1* observed in *bcr1AA* cells could account for their phenotypic defects. Finally, the phosphomimetic *bcr1EE* allele partially rescued the biofilm defects of *cbk1Δ* cells. This partial rescue could be taken as an indication that Cbk1 has additional functions in biofilm development that are independent of the two Bcr1 phosphorylation sites (T191 and S556). One possibility could be that Cbk1 also plays a role in regulating the Ace2 transcription factor. In *S. cerevisiae*, Cbk1-dependent phosphorylation of Ace2 is required for its biological function [20]. In *C. albicans*, expression of Ace2 target genes depends on Cbk1 [17] and we and others have shown that Ace2 is required for adhesion to surfaces [25]. Alternatively, it is also possible that Cbk1 controls other regulatory factors required for biofilm development. This is based on the fact that *ALS3* expression levels in *bcr1EE cbk1Δ* cells were still 2.6-fold lower than those of *bcr1EE* or *BCR1/bcr1Δ* cells (Figure 5D), suggesting that full expression of *ALS3* also requires Cbk1 in a Bcr1-independent manner. One possible scenario could be that Cbk1 also negatively regulates a repressor of *ALS3* expression, such as Nrg1 that contains three Cbk1 consensus sites (S200, T251 and T281). The transcriptional repressor Nrg1 downregulates *ALS3* transcription by binding to two regions in the *ALS3* promoter [35]. Thus, it is possible that full activation of *ALS3* expression during biofilm formation would require the inactivation of the Nrg1 repressor and the activation of the Bcr1 transcription factor, and that both events could be dependent on the phosphorylation of their Cbk1-consensus sites.

Another likely target of Cbk1 is the RNA-binding protein Ssd1 which contains multiple Cbk1-phosphorylation sites. In *S. cerevisiae*, the absence of Cbk1 in cells expressing functional Ssd1 severely impairs cell growth [36]. Recently, it has been shown that Ssd1 associates with specific mRNAs which encode proteins involved in bud growth and cell wall remodeling [37]. Cbk1-dependent phosphorylation of Ssd1 is required for translation of these mRNAs whereas Cbk1 inhibition promotes Ssd1 association with P bodies and thereby translational repression of Ssd1-associated mRNAs [37], [38]. Given the growth defect of *S. cerevisiae cbk1Δ* cells is suppressed by deletion of *SSD1*, a functional Ssd1 is likely to cause a negative effect on cell growth that is inactivated by Cbk1. In *C. albicans*, the same genetic interaction between *CBK1* and *SSD1* has been described [16]. Therefore, the activation of Ssd1 could account for the biofilm defects shown by the RAM mutants. However, since expression of Bcr1EE is able to alleviate the biofilm defects of *cbk1Δ* cells, it is likely that Cbk1 controls biofilm formation, at least partially, through the regulation of Bcr1 transcriptional activity. Further work on the functional interaction between Cbk1 and Ssd1 is an interesting area to determine the role of translational control during biofilm formation in *C. albicans*.

Regulation through phosphorylation is an important mechanism for controlling the activity of transcription factors [39], [40]. How Cbk1-dependent phosphorylation of Bcr1 regulates the transcriptional activity of the protein is unclear. In metazoans NDR/LATS kinase-dependent phosphorylation of YAP/TAZ co-activators results in their cytoplasmic retention and subsequent ubiquitination and degradation [23], [24], [41], [42]. In *S. cerevisiae*, the NDR/LATS kinase Cbk1 has the opposite functional output, since it promotes nuclear accumulation of the transcription factor Ace2 by phosphorylating two sites within its nuclear export sequence (NES) [20]. In *C. albicans*, our findings indicate that Cbk1 is not involved in regulating Bcr1 stability. Since Bcr1 does not contain an obvious NES close to the Cbk1 phosphorylation sites, we speculate that nuclear-cytoplasmic shuttling may not be a major output of Cbk1 phosphorylation. Therefore, a possibility is that the Cbk1-dependent phosphorylation might regulate the interaction of Bcr1 with other components of the transcription machinery.

Another significant conclusion of this work is that Cbk1-dependent regulation of Bcr1 is important for virulence in *C. albicans*. As previously described [29], we found that cells lacking Bcr1 were less virulent in disseminated murine candidiasis. Supporting a role for Cbk1 in Bcr1 activation, the *bcr1AA* strain showed a similar reduced virulence. Strikingly, the phosphomimetic *bcr1EE* mutant also showed the same reduced virulence. Since *C. albicans* mutants that are locked in the yeast form are less virulent in disseminated murine candidiasis [43]–[45], one possibility is that the virulence defect of these mutants might be a consequence of a defect in hyphal formation. However, *bcr1Δ* cells [8] and *BCR1* phosphomutants produced wild-type hyphae in hyphae-inducing conditions (not shown). Therefore, our results indicate that not only Cbk1-dependent phosphorylation of Bcr1, but also its dephosphorylation at specific moments of the infection process, are required for full virulence in *in vivo* models. Given that the addition of phosphates to specific residues of proteins can modify their interaction with other proteins [46], [47], a dynamic Bcr1 phosphorylation state could be required to modify its interaction with other regulatory factors required for Bcr1-dependent gene expression. This could aid in the colonization of different niches in response to environmental cues within the host. In *C. albicans*, developmentally regulated genes appear to be controlled by complex interactions between several transcription factors at their promoters [35], [48]. The *ALS3* gene is a good example of such complexity, since its expression is regulated by multiple transcription factors, including Efg1, Cph1, Bcr1, Nrg1, Rfg1 and Tup1 [35]. However, we could not exclude that the virulence defects observed in phosphomimetic *bcr1* mutants were due to conformational changes that might reduce its function during infection.

In sum, our results indicate that the RAM signaling pathway plays an important role in regulating Bcr1 function through its phosphorylation at two specific residues mediated by the Cbk1 kinase and that this phosphorylation is required for proper biofilm formation and virulence. Interestingly, the two phosphorylation sites are also conserved in other species, such as *Candida dubliniensis or Candida tropicalis*. In the *Candida parapsilosis* Bcr1 ortholog, which is also required for biofilm formation [49], the putative Cbk1 phosphorylation site at the end of the first Zn finger domain (T214) is also present. Therefore, the role of the RAM pathway in the control of Bcr1 function might be conserved in other biofilm-forming species.

## Materials and Methods

### Ethics statement

All animal experiments adhered to the EU Directive 86/609 on the approximation of laws, regulations and administrative provisions of Member States regarding the protection of animals used for experimental and other scientific purposes, and to related national regulations. All experiments were performed according to the guidelines of the European Convention for the Protection of Vertebrate Animals Used for Experimental and Other Scientific Purposes (ETS No. 123). The protocol was approved by Institut Pasteur Health Center Animal Care Committee (Protocol number: 10.455). Recovery of organs was performed following euthanasia of animals, and all efforts were made to minimize suffering.

### Media

*C. albicans* strains were grown in YPD (2% Bacto Peptone, 2% dextrose, 1% yeast extract) for transformation experiments. Transformants were selected on synthetic medium (2% dextrose, 6.7% YNB and auxotrophic supplements) or YPD supplemented with clonNAT at a final concentration of 200 µg ml^−1^ (Werner BioAgents) for Nat+ strains. For adherence and biofilm formation assays using microfermentors, overnight cultures were grown in synthetic medium with 0.4% dextrose supplemented with arginine, histidine and uridine (20 µg ml^−1^) and then diluted in the same medium supplemented with methionin (200 µg ml^−1^). For the biofilm formation on silicone squares, cells were grown in synthetic medium with 0.9% dextrose and supplemented with arginine, histidine, uridine and methionin at the concentrations mentioned above.

### Plasmid and strain construction

The strains used in this study are listed in Table 1 and derived from the BWP17 strain [50]. Disruption and epitope-tagged strains were made according to the PCR-mediated system using pFA plasmids [51], [52]. Strains were confirmed by PCR. Oligonucleotides were obtained from Biomers.net (Ulm, Germany) and are listed in Table 2. For site directed mutagenesis, fragments carrying *bcr1* T191A/E or S556A/E modifications were generated by PCR from genomic DNA of the JC1084 strain. The PCR products were inserted into pSC-A vector (Strataclone PCR cloning kit) for sequencing and then digested with both *Eco*RV and *Cla*I for the T191 fragment or *Bst*BI for the S556 fragment, giving rise to a DNA fragment of 798 bp or 666 bp, respectively. These fragments were used to swap the wild-type *BCR1* regions of a pGEM-*URA3*-based vector, that contained the complete wild type *BCR1* ORF fragment cloned into the *Sac*I site and a 3′UTR–*BCR1* region inserted into the *Not*I site (+194 to +704 from the stop codon) to direct integration of the plasmid to the *BCR1* locus. The constructions were digested with *Sal*I and *Afl*II and transformed into a *BCR1* heterozygous strain using a standard lithium acetate method [53]. The transformants were confirmed by PCR and sequencing. Strains used for biofilm formation assays were transformed to uracil and histidine prototrophy using *Stu*I-linearized CIp10 [54] or *Sac*I/*Sac*II-digested ECC72 plasmids respectively. ECC72 contains 2.9 kb from the *HIS1* locus (from 1000 bp upstream of the start codon to 1000 bp downstream of the stop codon) which were PCR amplified and cloned in the pGEM-T (Promega) *Eco*RV site.

**Table 1:** Strains used in this study.

Name	Genotype	Source
CEC369	*ura3Δ::λimm434/ura3Δ::λimm434 arg4Δ::hisG/ARG4 his1Δ::hisG/HIS1 RPS1/rps1::CIp10-URA3*	[55]
JC305	*CBK1-myc::HIS1/CBK1*	[17]
JC343	*ace2*::*ARG4*/*ace2*::*URA3 CBK1-MYC-HIS1/CBK1*	This study
JC525	*mob2*::*ARG4*/*mob2*::*URA3 CBK1-YFP-HIS1/CBK1*	This study
JC798	*kic1*::*ARG4*/*kic1*::*HIS1 MOB2-HA-URA3/MOB2*	This study
JC848	*tao3*::*ARG4*/*tao3*::*HIS1 MOB2-HA-URA3/MOB2*	This study
JC1080	*cbk1*::*ARG4*/*cbk1*::*HIS1 RPS10/rps10::CIp10-URA3*	This study
JC1081	*bcr1::URA3*/*bcr1*::*ARG4 his1Δ/HIS1*	This study
JC1082	*bcr1::BCR1-URA3*/*bcr1*::*SAT1 cbk1*::*ARG4*/*cbk1*::*HIS1*	This study
JC1088	*bcr1-S556A-URA3/bcr1*::*ARG4 his1Δ/HIS1*	This study
JC1089	*bcr1::BCR1-URA3*/*bcr1*::*ARG4 his1Δ/HIS1*	This study
JC1092	*bcr1-S556E -URA3*/*bcr1*:: *ARG4 his1Δ/HIS1*	This study
JC1093	*bcr1-T191A-URA3*/*bcr1*::*ARG4 his1Δ/HIS1*	This study
JC1094	*bcr1-T191E-URA3*/*bcr1*::*ARG4 his1Δ/HIS1*	This study
JC1096	*bcr1-T191A/S556A-URA3*/*bcr1*::*SAT1 cbk1*::*ARG4*/*cbk1*::*HIS1*	This study
JC1099	*bcr1-T191E/S556E-URA3*/*bcr1*::*SAT1 cbk1*::*ARG4*/*cbk1*::*HIS1*	This study
JC1144	*BCR1-HA::URA3*/*bcr1*::*ARG4 his1Δ/HIS1*	This study
JC1151	*BCR1-HA::URA3*/*bcr1*::*ARG4 CBK1-myc::HIS1/CBK1*	This study
JC1159	*BCR1-HA::URA3*/*BCR1 cbk1*::*ARG4*/*cbk1*::*HIS1*	This study
JC1176	*bcr1-T191A/S556A-HA::SAT1-URA3/bcr1*::*ARG4 his1Δ/HIS1*	This study
JC1177	*bcr1-T191E/S556E-HA::SAT1-URA3/bcr1*::*ARG4 his1Δ/HIS1*	This study
JC1178	*bcr1-T191A/S556A-URA3/bcr1*::*ARG4 his1Δ/HIS1*	This study
JC1180	*bcr1-T191E/S556E-URA3/bcr1*::*ARG4 his1Δ/HIS1*	This study

All the strains referred in this work derive from BWP17.

**Table 2:** Primers used in this study.

Deletion and gene disruption	
Name	Sequence 5′-3′
S1*BCR1*	GAATCATTCATTCATTCTAATTGTTGGGATATTTTATTTTATCAAGTTTTTATAATAATACAAATCTATCAATATTATTT AATAATAACTTAAATTTTCATTGAAGCTTCGTACGCTGCAGGTC
S2*BCR1*	AACAAATAGTATATATGTAAATCAAGTAGAACACTCATACTCAGTTTATATAACAAACGAGTAAAGTAAGAACACTATAA AAAAGAAACAACATCAAAAATCTGATATCATCGATGAATTCGAG

Tagging	
Name	Sequence 5′-3′
S1*BCR1*HA	AACAACAACAAATACAAATACAAATACAAATAGTACGGCTACTACTAATGCTACTGGGTATGTTTCGAAAAAACCTACGATAAATAATTTAATATCACAAACGCGTTATCCATATGATGTTCC
S2*BCR1*HA	AACAAATAGTATATATGTAAATCAAGTAGAACACTCATACTCAGTTTATATAACAAACGAGTAAAGTAAGAACACTATAAAAAAGAAACAACATCAAAAACCAGGGTTTTCCCAGTCACGACG
S1*CBK1myc*	TGCTAAGAATGGAGGCGGCAGAAAGAATCCAAAGGAAGATTTACCATTTATTGGATACACTTATTCTAGATTTGATTATTTGACA AGAAAGAATGCG TTACGGATCCCCGGGTTAATTAA
S2*CBK1myc*	ATAATGCATAAACAATAACATCATCCGGCTGTACTACCATTCCAAATGACCACCTATTGTTGATACATGTATGATAAGAAAAGGT TGGCATGCTTTGTTGGAATTCCGGAATATTTATGAGAA

Bcr1 site-directed mutagenesis	
Name	Sequence 5′-3′
M1-*BCR1*-SacI	AGCTCGAGCTCATCATGTCAGGGACATCACAAGTACTTC
M2-*BCR1*-SphI	AGCTCGCATGCATCAAGTAGAACACTCATACTCAGT
M1B-*BCR1*	TCTAATGAAAATATTAGTAATGAAGGG
M2B-*BCR1*	CTAGGTGGTGGTGGATAAACTGCAT
M3A *BCR1*	CTCGTCATAAACGTAAACATGCGGGTGAACGACCTTTTACTTG
M3E *BCR1*	CTCGTCATAAACGTAAACATGAGGGTGAACGACCTTTTACTTG
M4A *BCR1*	CAAGTAAAAGGTCGTTCACCCGCATGTTTACGTTTATGACGAG
M4E *BCR1*	CAAGTAAAAGGTCGTTCACCCTCATGTTTACGTTTATGACGAG
M5A *BCR1*	CTTTTCATCAACGTCAAAGTGCGGTTGTTAGTGATTTTCTGAT
M5E *BCR1*	CTTTTCATCAACGTCAAAGTGAGGTTGTTAGTGATTTTCTGAT
M6A *BCR1*	ATCAGAAAATCACTAACAACCGCACTTTGACGTTGATGAAAAG
M6E *BCR1*	ATCAGAAAATCACTAACAACCTCACTTTGACGTTGATGAAAAG
3′UTR-*BCR1*-5′	AGCTCTCTAGAGGGGTGACGACAGTGTTTAATTTAAT
3′UTR-*BCR1*-3′	AGCTCGCGGCCGCATTGAAATTCAACAATCTCCCTTCCCA

Sequencing	
*BCR1*-1S	CACAGCACGCATCTATGGCTTATAA
*BCR1*-2S	CACTAACGCCGACATTAACCAATGCTAC
*BCR1*-3S	GCTTATGAAACTTATTTAACTAAGGATAATCG
*BCR1*-4S	AGGTTATTTGGATCCATATAGACAA
*BCR1*-5S	CCACCACCACCTAGATCAGCAACTT
*BCR1*-6S	GAGCAACAACAACAACAACTACAAG
*BCR1*-7S	CGGCTACTACTAATGCTACTGGGTA

Integration	
Name	Sequence 5′-3′
G1*BCR1*	CCCCATATTACATATCATATCTTTA
G2*BCR1*	CCGCCCCCCCTTACACGACACACCG
G1*CBK1myc*	ACAAACCATGCAAACATGGAG
G1*CBK1*	CACTTCAAAGCAATTTGAGAATACGCCTCC
G2*CBK1*	GGTCATTCAAGGGTGAGCAAG
*MYC2*	CACCGTCGAGTCCGTTCAAGTC
X2Ca*ARG4*	AATGGATCAGTGGCACCGGTG
X3Ca*ARG4*	GCTCTTGGTGGTACTGCTAAAAGTGCCG
X2Ca*HIS1*	CAACGAAATGGCCTCCCCTACCACAG
X3Ca*HIS1*	GACGAATTGAAGAAAGCTGGTGCAACCG
X2Ca*URA3*	GTGTTACGAATCAATGGCACTACAGC
X3Ca*URA3*	GGAGTTGGATTAGATGATAAAGGTGATGG
X2*SAT1*	GCACACACTACTTAATATACACAG
X3*SAT1*	GTGAAGTGTAAGGGGGAG

Quantitative RT-PCR	
Name	Sequence 5′-3′
*ADE2* forward	TGTTGTCACATCTTCCATGC
*ADE2* reverse	ATTCCCACCAATGGAGATTC
*ALS1* forward	AGCTTGACAACAGGCACCTC
*ALS1 reverse*	TCATTTGAAGCACTGGCAAC
*ALS3* forward	CGTCCTCATTACACCAACCA
*ALS3* reverse	ACAACCTTTATCACCTTCACAGC

### Adherence

Surface adherence was determined incubating plastic slides (Thermanox, Nunc) with OD_600_ = 1 cell cultures during 1 hour at room temperature and washing three times with PBS to remove non-adherent cells. Cells attached to the plastic surface were recovered by vortexing and resuspended in 15 µl of PBS, using 5 µl for counting the adherent cells with an optical microscope. For each strain the average of cells/field is the result of counting a total of 30 fields from three independent experiments.

### Biofilm formation

Microfermentor assays were done as described previously [7] while the method of the silicone squares used for microscopy analysis was described in [12]. For dry mass measurements, after the incubation period in the microfermentor, the plastic slides were removed from the glass spatula and transferred to a 50 ml tube with 10 ml of water. Cells were detached from the plastic surface by vortexing and then recovered by filtration using Millipore 1.2 µm filters. Filters were dried during 48 hours at 60°C prior mass measurement. Total biomass of each biofilm sample was calculated by subtracting the mass of a blank filter subjected to the same washing and drying treatment. For each strain the experiment was repeated twice in duplicate.

### Microscopy and image analysis

Biofilm development in microfermentors was recorded with a Nikon Coolpix digital camera. For the silicone square experiments, biofilms were observed by CSLM, after staining with calcofluor white 0.01% (vol/vol) and 10 µg ml^−1^ concanavaline A Alexa fluor 594 conjugate (Invitrogen) for 1 h in the dark at 37°C with 150 rpm agitation. CSLM was performed with an upright Zeiss Axioskop2 FS MOT LSM 510 multiphoton microscope using a Zeiss Achroplan ×40/0.8 W objective. All CSLM stacks were assembled into projections using the Image J software.

### Protein extracts, Western Blotting, 2D-WB and immunoprecipitation

Cells extracts, Western blotting, 2D-WB and immunoprecipitation assays were done as previously described [17].

### RNA isolation and expression analysis

Cultures for RNA extraction were done as previously described [9]. For RNA extraction mid-logarithmic cell cultures were harvested, frozen in liquid nitrogen and stored at −80°C. 50 mg of cells were lysed in a FastPrep cell disruptor in the presence of 50 µl TRIzol Reagent (Invitrogen). Total RNA was isolated according to manufacturer's instructions. The quantity, quality and integrity of the RNA were analyzed in an Agilent's 2100 Bioanalyzer system. cDNA synthesis was performed with the SuperScript II First-Strand Synthesys System (Invitrogen) using oligo(dT), from 3 µg total RNA previously treated with DNAase I (Invitrogen). qPCR assays were done using SYBR Premix Ex Taq (TaKaRa). For each reaction, 1 µl cDNA was used. No-template and no-reverse transcription controls were included. The assays were carried out in duplicates at generic cycle conditions (95°C for 45 s and 40 cycles of 95°C for 5 s and 60°C for 31 s, followed by a dissociation step at 95°C for 15 s, 60°C for 1 min and 95°C for 15 s) in an Applied Biosystems 7300 Real-Time PCR System (Applied Biosystems). Relative gene expression quantification was achieved using the Pfaff1 method.

### Disseminated murine candidiasis assays

Nine week-old female Balb c/J mice were infected intravenously with 5×10^5^ CFU/mouse. Ten mice per strain were tested per experiment and mice were kept in groups of 5 per cage. 2 days after infection 2 mice/strain were sacrificed, the kidneys taken, homogenized and plated on SD medium containing 50 µg/ml ticarcillin and 10 µg/ml gentamycin. The fungal burden was determined by counting CFUs after 2 days at 30°C. Mice were checked 2 times per day for survival over a period of 14 days. At day 14 all remaining mice were sacrificed. Results were analyzed using the software GraphPad Prism5.
